# Evaluation of a surface imaging system's isocenter calibration methods

**DOI:** 10.1002/acm2.12054

**Published:** 2017-03-06

**Authors:** Adam B. Paxton, Ryan P. Manger, Todd Pawlicki, Gwe‐Ya Kim

**Affiliations:** ^1^ Department of Radiation Oncology University of Utah Huntsman Cancer Hospital Salt Lake City UT 84112 USA; ^2^ Department of Radiation Medicine and Applied Sciences University of California San Diego Moores Cancer Center La Jolla CA 92093 USA

**Keywords:** isocenter calibration, optical surface imaging, stereotactic radiosurgery

## Abstract

AlignRT is a surface imaging system that has been utilized for localizing and tracking patient position during radiotherapy. AlignRT has two calibration procedures that can set the system's isocenter called “Monthly Calibration” (MC) and “Isocentre Calibration” (IC). The MC utilizes a calibration plate. In addition to the calibration plate, the IC utilizes a cubic phantom that is imaged with the linac treatment beam to aid in aligning the AlignRT and treatment‐beam isocenters. This work evaluated the effects of misaligning the calibration plate during the calibration process. The plate was intentionally shifted away from isocenter ±3.0 mm in the longitudinal and lateral directions and ±1.0 mm in the longitudinal, lateral, and vertical directions. A mock stereotactic radiosurgery (SRS) treatment was used to evaluate the effects of the miscalibrations. An anthropomorphic head phantom was placed in an SRS treatment position and monitored with the AlignRT system. The AlignRT‐indicated offsets were recorded at 270°, 315°, 0°, 45°, and 90° couch angles for each intentional misalignment of the calibration plate during the MC. The IC was also performed after each miscalibration, and the measurements were repeated and compared to the previous results. With intentional longitudinal and lateral shifts of ±3.0 mm and ±1.0 mm of the calibration plate, the average indicated offsets at couch rotations of ±90° were 4.3 mm and 1.6 mm, respectively. This was in agreement with the theoretical offset of √2*(shift‐of‐the‐calibration plate). Since vertical shifts were along the rotation axis of the couch, these shifts had little effect on the offsets with changing couch angle. When the IC was applied, the indicated offsets were all within 0.5 mm for all couch angles for each of the miscalibrations. These offsets were in agreement with the known magnitude of couch walkout. The IC method effectively removes the potential miscalibration artifacts of the MC method due to misalignments of the calibration plate.

## 1 Introduction

AlignRT (Vision RT Ltd, London, UK) is a 3‐camera, non‐invasive, non‐radiographic, optical surface imaging system that provides the user with translational and rotational offsets from a reference surface as well as the total displacement (i.e., the vector sum of the translations).^1^ The reference surface can be obtained in two ways, either from a computed tomography (CT)‐defined body contour that has been imported from the treatment planning system (TPS) or from a reference image that is captured by the AlignRT system at the time of treatment when the patient is in the final treatment position.

AlignRT has two calibration procedures that can set the imaging system's isocenter. These are called “monthly calibration” (MC) and “isocentre calibration” (IC). The MC procedure involves placing a calibration plate centered at the linac isocenter to set the imaging system's isocenter. The IC procedure involves imaging a cubic phantom with implanted ceramic spheres with the linac treatment beam to aid in aligning the imaging system's isocenter with the treatment beam's isocenter.

AlignRT has been utilized for a number of treatment sites, including: breast,^2^, ^3^, ^4^, ^5^ extremities,^6^ head and neck,^7^, ^8^ and frameless stereotactic radiosurgery (SRS).^9^, ^10^ Due to the tighter tolerances typically required for SRS treatments and the use of couch rotations, this work focused on the SRS treatment procedure. However, the results are applicable to other treatment sites.

When using AlignRT with SRS, the patient is typically immobilized in an open‐face mask. Prior to treatment, a region‐of‐interest (ROI) is defined consisting of the patient's face. The typical workflow involves: initially aligning the patient based on room lasers, using AlignRT to finely adjust the patient position (translations and rotations) based on the reference CT‐defined body contour, using radiographic analysis (e.g., orthogonal kV image pair and CBCT) to shift the patient into the final treatment position, capturing a new reference surface with the AlignRT system, and monitoring the intrafractional motion during treatment based on this new reference surface.^9^, ^10^

For treatment plans that utilize couch rotations, like SRS, the planned couch angles are available in a dropdown list in the AlignRT user interface. Selecting a different couch angle will rotate the reference surface relative to the AlignRT isocenter and allow tracking the patient at these positions. However, misalignments of the linac treatment beam and AlignRT isocenters may propagate as falsely indicated offsets with the AlignRT system when couch rotations are used. This work investigated the effects of these potential misalignments between the treatment beam isocenter and the AlignRT isocenter for each of the isocenter calibration methods.

## 2 Materials and methods

### 2.A Calibration procedures

**Figure 1:**
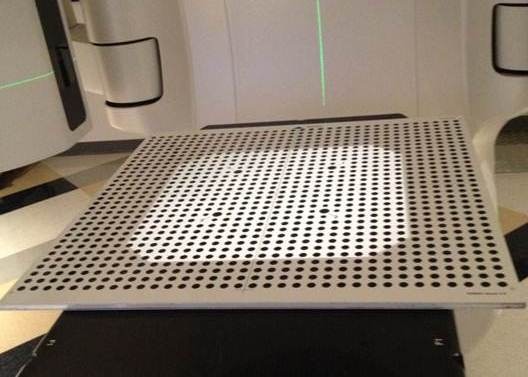
Photograph of the AlignRT calibration plate.

The MC procedure utilizes a calibration plate that is provided by the manufacturer (see Fig. 1). The plate consists of a 2D array of high‐contrast circles with known dimensions and location. Four of the circles are labeled with the numbers 1 to 4, allowing the different camera pods to correctly identify the orientation of the calibration plate. During the calibration process, the center of the calibration plate is placed as close to the linac isocenter as possible. The AlignRT manual suggests aligning the calibration plate cross‐hairs with the linac field cross‐hairs or the room lasers and placing the surface of the calibration plate at 100 cm source‐to‐surface distance (SSD). Next, the numbered circles are located on images taken from each of the three camera pods. This allows for a spatial correlation between the cameras that allows the system to triangulate objects in space. The AlignRT system's isocenter is set at the center of the cross‐hair on calibration plate.

**Figure 2:**
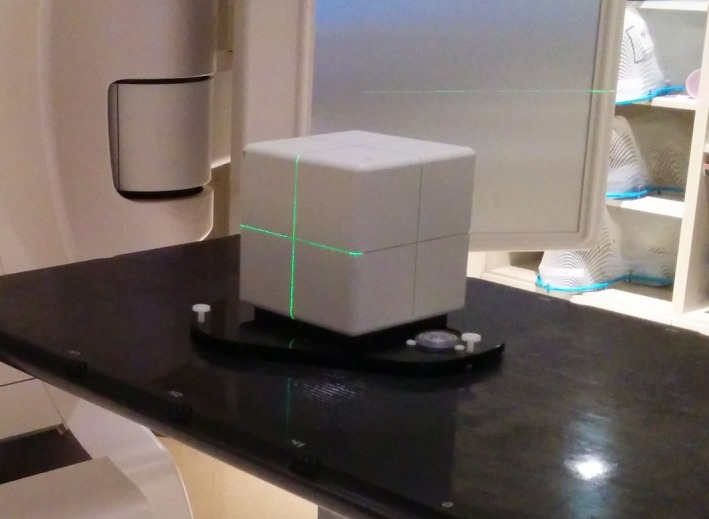
Photograph of the AlignRT isocenter calibration phantom. The cubic phantom has five embedded ceramic spheres used for localizing the phantom with MV imaging.

The IC procedure still requires the spatial calibration with the calibration plate, so the MC must still be completed. In addition, the IC uses a cube phantom with five‐embedded ceramic spheres (see Fig. 2). One of the spheres is located at the center of the cube, while the other four are arranged asymmetrically around the center sphere. During the calibration process, the phantom is leveled on its baseplate and positioned near the linac isocenter by aligning the phantom cross‐hairs with the room lasers and/or the linac field cross‐hairs. Four megavoltage (MV) portal images are acquired at gantry angles of 0°, 90°, 180°, and 270°. The images are imported into the AlignRT software and the IC module is started. The surface position of the cube is determined by momentarily monitoring the cube phantom with the AlignRT system. A radiographic analysis is then performed based on the location of the embedded spheres in the four MV portal images. The offsets from the determined linac treatment beam isocenter and the position of the cube are given. The user then applies the isocenter calibration of the AlignRT system. This calibration shifts the previous AlignRT isocenter determined during the MC with the calibration plate to the linac beam isocenter determined from the MV portal images of the isocenter calibration phantom.

### 2.B Head phantom

**Figure 3:**
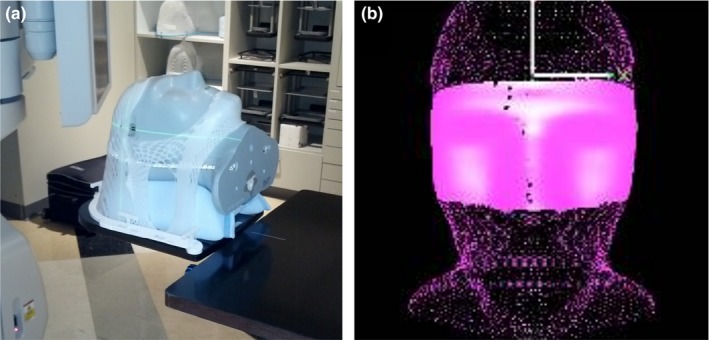
Images of the IMT MAX‐HD head phantom in the open‐face mask (a) and the ROI defined in AlignRT that includes areas not obscured by the immobilization mask (b).

For this work, a MAX‐HD anthropomorphic head phantom from Integrated Medical Technologies (IMT) (Troy, NY, USA) was used to closely replicate a patient SRS treatment setup. For example, a similar open‐face mask was used and AlignRT ROI was defined (see Fig. 3).

Similar to the actual patient procedure, the head phantom was CT simulated in a custom foam headrest and in an open‐face mask with 1.25 mm slice thickness. The CT images were imported into Eclipse TPS (Varian Medical Systems, Palo Alto, CA, USA). A mock treatment plan was created with isocenter positioned on an internal marker in the head phantom, roughly centered in the brain. The treatment plan included fields with couch rotations of 0°, 45°, 90°, 315°, and 270°.

The treatment plan and body structure were imported into AlignRT and an ROI of the open areas of the phantom's face were defined as shown in Fig. 3(b). The head phantom was initially positioned using kV CBCT imaging at the linac. This work was completed on a TrueBeam linac (Varian Medical Systems, Palo Alto, CA, USA) for which the Varian IsoCal calibration procedure had been performed.

### 2.C Displacements with intentional miscalibrations

To evaluate the potential problems with the calibration method that only utilizes the calibration plate, intentional miscalibrations were applied. Using table shifts, the calibration plate was displaced in one direction from the linac isocenter prior to the MC to cause a miscalibration. The one‐dimensional miscalibrations investigated were: ±3.0 mm in the longitudinal (lng) and lateral (lat) directions and ±1.0 mm in the vertical (vrt), lng, and lat directions. This resulted in a total of 10 investigated miscalibrations. After each miscalibration, the head phantom was returned to the position initially indicated using CBCT. AlignRT surface tracking was started using the CT‐defined reference surface and the indicated offsets were recorded at each of the couch rotational positions from the treatment plan.

In addition, to investigate the procedure used clinically for SRS, a new reference surface was captured with AlignRT with the head phantom at the position initially indicated using CBCT (at a couch rotation of 0°). Surface tracking was started and again, the couch was rotated to the planned couch rotations and the indicated displacements were recorded.

### 2.D Displacements after isocenter calibration

Following each of the intentional miscalibrations with the calibration plate during MC, an IC with the cube phantom was completed. Again the head phantom was returned to the position initially indicated by CBCT. A new reference image was captured with AlignRT and the couch was rotated to the planned positions and the indicated displacements were recorded. These results were compared to the results prior to the IC.

## 3 Results

### 3.A Displacements with intentional miscalibrations

**Figure 4:**
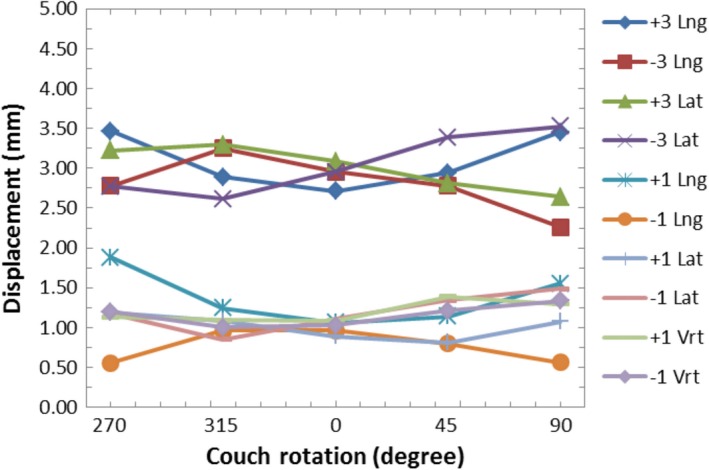
Graph of the AlignRT‐indicated displacements vs. couch rotation while using the CT body‐defined reference surface for various intentional miscalibrations.

A graph of the AlignRT‐indicated displacements vs. couch angle while using CT body‐defined reference surface for various intentional miscalibrations is shown in Fig. 4. For this setup, the displacement magnitude was approximately constant with couch rotations as the plots for each of the miscalibrations are flat in shape. The average displacements for 3.0 mm and 1.0 mm intentional miscalibrations were 3.0 mm and 1.2 mm, respectively. So the indicated displacement was approximately equal to the miscalibration magnitude.

**Figure 5:**
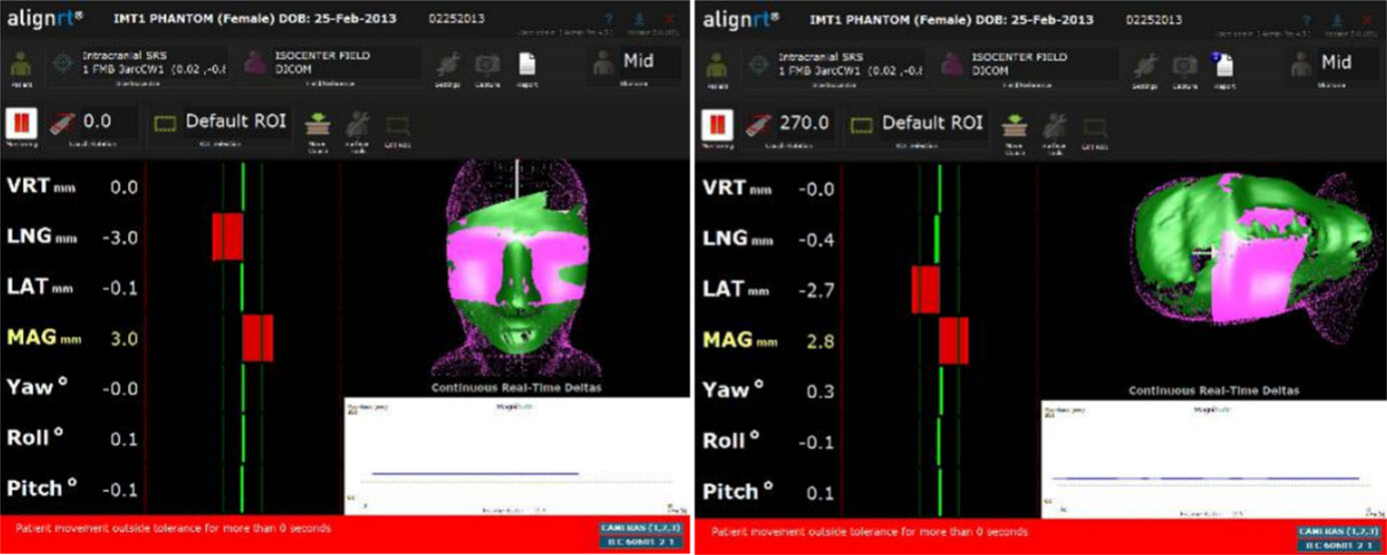
The AlignRT display of the head phantom relative to the CT‐defined reference surface with a −3.0 mm lng intentional miscalibration at a couch angle of 0° (left) and at 270° (right).

Figure 5 shows AlignRT screenshots of the head phantom relative to the CT‐defined reference surface after a −3 mm lng miscalibration. The indicated offsets are −3.0 mm in the lng direction with the couch at 0°. At a 270° couch rotation, the total displacement remains roughly constant, only changing from 3.0 mm to 2.8 mm; however, the direction of the displacement changes from lng to lat. That is, the direction of the indicated offset remains in the same direction relative to the treatment room.

**Figure 6:**
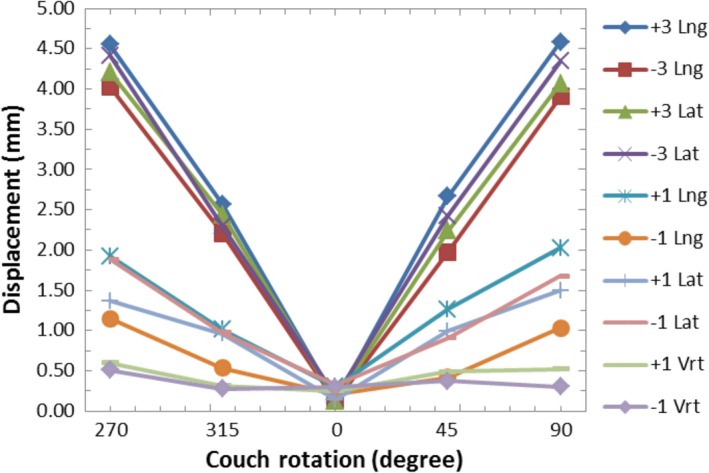
Graph of the AlignRT‐indicated displacements vs. couch rotation while using the AlignRT‐acquired reference surface for various intentional miscalibrations before the isocenter calibration was applied.

A graph of the AlignRT‐indicated displacements vs. couch angle while using the AlignRT‐captured reference surface for various intentional miscalibrations is shown in Fig. 6. Again this is representative of the workflow that would be used clinically for SRS, where a new reference surface would be acquired with AlignRT with the couch at 0°. For this setup, the displacement magnitude was approximately 0 mm at 0° couch angle as the average displacement was 0.2 mm for all intentional miscalibrations. As the couch was rotated, the indicated displacement increased for miscalibrations in the lng and lat directions. The rate of increase in the indicated displacement was greater for larger miscalibrations. The average displacement at couch angles of 90° or 270° was 4.3 mm for ±3.0 mm miscalibrations in the lng or lat directions and 1.6 mm for ±1.0 mm miscalibrations in the lng or lat directions. For miscalibrations in the vrt direction, the plot of displacement vs. couch rotation had a flat shape. The largest indicated displacement was 0.6 mm (Fig. 6).

**Figure 7:**
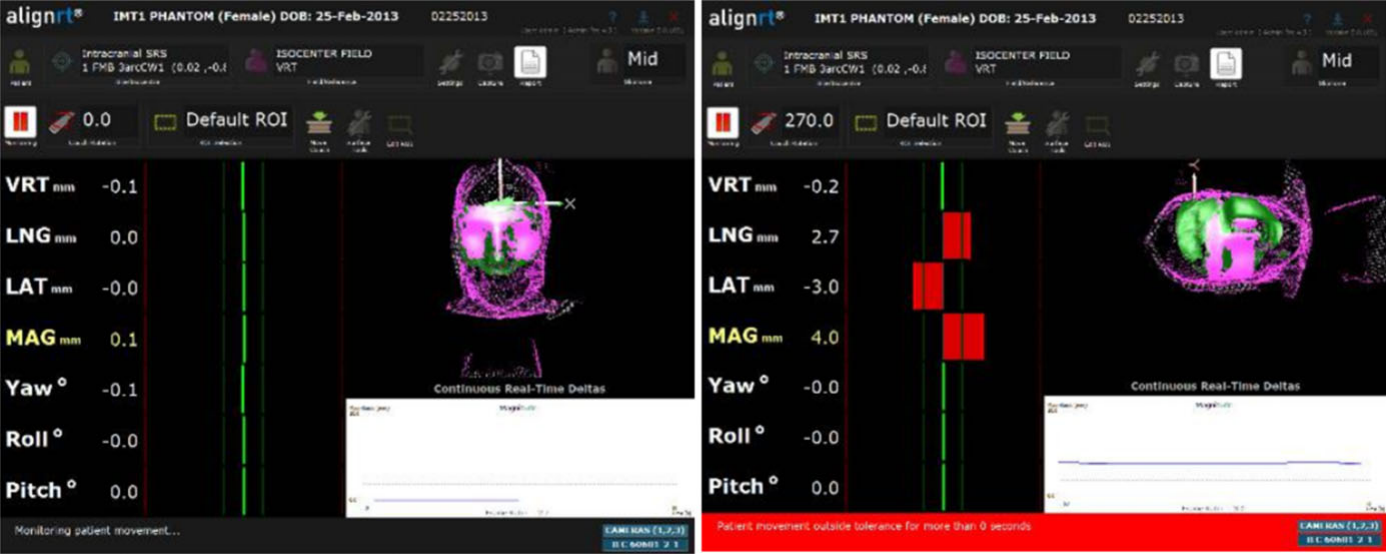
The AlignRT display of the head phantom relative to the AlignRT‐acquired reference surface with a −3.0 mm lng intentional miscalibration at a couch angle of 0° (left) and at 270° (right).

Figure 7 shows AlignRT screenshots of the head phantom relative to the AlignRT‐acquired (at 0° couch angle) reference surface after a −3.0 mm lng miscalibration. The indicated offsets are approximately zero at a couch angle of 0°. However, at a couch angle of 270°, the offsets increase in both the lat and lng directions resulting in a total indicated displacement of 4.0 mm.

**Figure 8:**
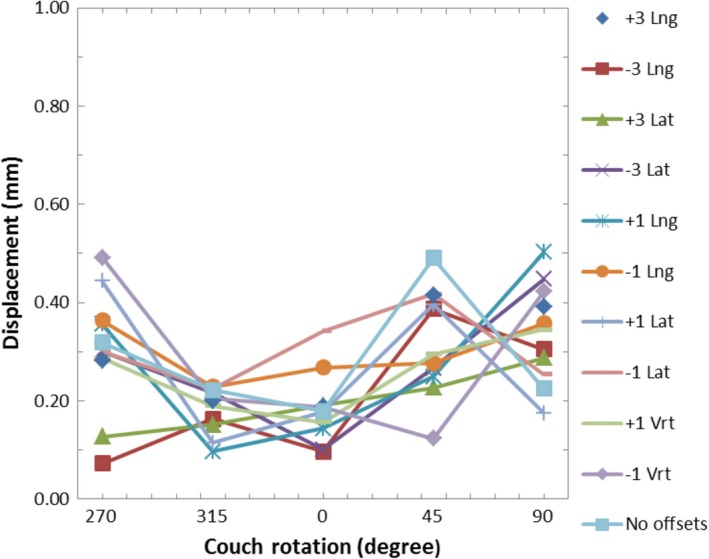
Graph of the AlignRT‐indicated displacements vs. couch rotation while using the AlignRT‐acquired reference surface for various intentional miscalibrations after the isocenter calibration was applied.

### 3.B Displacements after isocenter calibration

A graph of the AlignRT‐indicated displacements vs. couch angle while using the AlignRT‐captured reference surface for various intentional miscalibrations after IC is shown in Fig. 8. Note the different scale of the displacement axis relative to Figs. 4 and 6. The plots for all of the miscalibrations have a flat shape. The AlignRT‐indicated displacements ranged from 0.1 mm to 0.5 mm. These values are within the expected range of walkout observed for this couch from ongoing quality assurance (QA) tests.

## 4 Discussion

In theory, if the MV beam and AlignRT isocenters are perfectly aligned, the increase in AlignRT‐indicated displacements during couch rotations when using the AlignRT‐captured reference surface should only be due to couch walkout (assuming the patient has not moved and the AlignRT system is able to accurately visualize the patient at the various couch rotations). If this is the case, the user could correct the position of the patient to account for the couch walkout at each couch rotation. This would ensure the patient was always aligned to the MV beam isocenter, and the strict mechanical tolerances typically required of the couch to preform SRS treatments may no longer be as vital. This work did not test the feasibility of using AlignRT for this purpose, but evaluating the ability of the isocenter calibration of the system is the first step toward being able to investigate this feasibly. Additional work would need to confirm that there are no AlignRT‐indicated displacement artifacts due to the patient being rotated and the resulting change in the areas of the ROI that are visible to the AlignRT cameras. In addition, changes in the load on the couch and its position relative to the couch pedestal could affect the magnitude of the couch walkout. For this investigation, the routine QA results that were used to estimate the expected couch walkout had a similar load on the couch compared to this study. However, patients apply different weight loads to the couch, which could alter the magnitude of couch walkout. These potential effects would need to be investigated.

**Figure 9:**
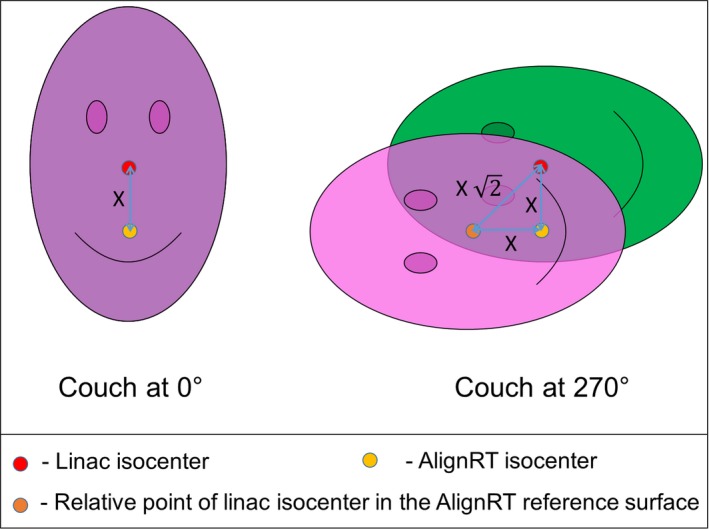
Image demonstrating the geometric effect of an isocenter misalignment of distance X between the linac and AlignRT system when an AlignRT‐acquired reference surface is used. With the couch at 0°, the reference and monitored surface match. However, when the couch is rotate ±90°, the magnitude of the indicated offset between the reference and monitored surface is given as (X √2).

As shown in Fig. 6, miscalibrations of the AlignRT isocenter in the lng or lat direction cause the AlignRT‐indicated offsets when using the AlignRT‐acquired reference surface to show false values when the couch is rotated. Based only on geometry, one would expect the AlignRT‐indicated displacement at ±90° couch rotations to be equal to √2*(the magnitude of the miscalibration) for lng and lat miscalibrations. This is visually demonstrated in Fig. 9. For 3.0 mm and 1.0 mm miscalibrations, this would be 4.2 mm and 1.4 mm, respectively. For this work, the average displacements for 3.0 mm and 1.0 mm lng or lat miscalibrations were 4.3 mm and 1.6 mm, respectively. The observed values are expected to be slightly larger as they include couch walkout.

As seen in Fig. 6, the indicated displacements with miscalibrations in vrt direction are within 0.6 mm. Miscalibrations in the vrt direction are along the axis‐of‐rotation the couch and so do not create false readings as the couch is rotated. The 0.6 mm max value is within the expected range of couch walkout.

Based only on geometry, an offset in the calibration plate of 0.7 mm (√2/ 2) in the lat or lng directions from the linac beam isocenter would result in an falsely indicated offset of 1.0 mm at 90° couch rotations. This would reach the 1.0 mm threshold often used for SRS treatments^10^ from the isocenter misalignment artifact alone. Placing the calibration board with submillimeter accuracy may be difficult when using the room lasers or other surrogates for isocenter.

As seen in Fig. 8, the IC process resolves calibration plate alignment issues. The observed displacements with couch rotations were all within 0.5 mm, which is within the range expected due to couch walkout.

Although comparison of the AlignRT‐indicated offsets when using the CT‐defined reference surface with intentional miscalibrations may not be relevant for SRS treatments (since a new reference image is typically captured after image‐guided shifts have been completed), it is relevant for other utilizations of the AlignRT system that may use the CT‐defined body as a reference surface for the initial setup of the patient. An example would be deep‐inspiration breath hold (DIBH) for the treatment of left‐sided breast or chest wall.^2^, ^3^, ^4^ In this setting, a miscalibration (without subsequent isocenter calibration) would result in a systematic offset in the reference surface. This is effect is demonstrated by the data displayed in Fig. 4. However, treatments such as DIBH for left‐sided breast do not typically require the submillimeter setup tolerance required by SRS treatments, so performing only the MC with the calibration plate could be sufficient for these treatments provided the calibration plate was setup very carefully. According to Task Group 142, the suggested mechanical tolerance for the ODI and room lasers (for non‐IMRT machines) is 2 mm.^11^ To limit the effects demonstrated in Fig. 4, AlignRT users should use the front pointer (rather than the ODI) for setting the SSD to the calibration plate and the linac cross‐hair for aligning the calibration plate cross‐hair. If the lasers are going to be used for aligning the calibration plate, their correct positioning should be confirmed beforehand. The levelness of the calibration plate should also be confirmed prior to performing the MC to prevent systematic pitch or roll offsets.

A limitation of using a plastic phantom to evaluate the effects of miscalibrations with the CT‐defined body as the reference surface is that the phantom does not exactly emulate human skin. The AlignRT‐indicated offset from the CT‐defined reference surface may be slightly different between a phantom and human skin. Even between patients, there could be variations in the AlignRT‐indicated offset depending on the CT number used to define the body structure, skin tone, or other physical properties of the skin. Regardless of the potential differences between the phantom and human skin, the presented results remain appropriate in at least informing the users of the AlignRT system of the effects that could be seen with isocenter miscalibrations when using the CT‐defined body as the reference surface.

The IC is most valuable for treatments that utilize couch rotations and have tight tolerances. Presently the IC is recommended for the AlignRT system when used for SRS brain treatments. Recent research has found benefits in utilizing non‐coplanar beams in stereotactic body radiation therapy (SBRT) treatments of lung,^12^ liver,^13^ and head and neck^14^ sites. AlignRT has already been utilized to track the position of patients being treated for head and neck cancers^8^ in a manner that was similar to SRS. That is, an open‐faced masked was used and the ROI was defined as the patient's face. AlignRT users should evaluate the potential need for the IC before utilizing the system for these non‐coplanar SBRT treatment techniques.

It is worth noting that an offset in calibration plate (intentional or not) during the daily QA of the AlignRT system will not result in a displacement of the AlignRT isocenter since the daily QA is simply confirming the spatial correlation between each of the camera pods is still intact (within a tolerance). In addition, the MC has the option to take additional images of the calibration plate raise 75 mm from isocenter. Again, this does not affect the isocenter of the AlignRT system, but is used to help the accuracy of the system in the situation where the isocenter is located posteriorly in the patient which results in the monitored surface being farther from isocenter.

## 5 Conclusions

This work evaluated both the MC and IC methods for positioning the AlignRT isocenter. The potential pitfalls of the MC method that relies on manual placement of the calibration plate for positioning the AlignRT system's isocenter were demonstrated. The advantages of the IC method that utilizes the linac treatment beam with the cubic isocenter phantom were established. The IC for the AlignRT system provides better coincidence of the imaging isocenter with linac beam isocenter. This effectively removes the potential for miscalibration artifacts that can be seen during couch rotations. This is especially critical for treatment methods that have tight tolerances and utilize couch rotations, such as SRS. The IC method is less critical for treatments that do not use couch rotations or do not require submillimeter accuracy, provided that the MC method with the calibration plate was performed carefully.

## Conflict of Interest

The authors have no conflicts of interest.
